# Multi-frequency dielectrophoretic characterization of single cells

**DOI:** 10.1038/s41378-018-0023-4

**Published:** 2018-09-10

**Authors:** Alex Jaffe, Joel Voldman

**Affiliations:** Department of Electrical Engineering and Computer Science, Massachusetts Institute of Technology, Cambridge, MA USA

## Abstract

We explore the use of dielectrophoresis to discern the electrical properties of single cells by observing them at multiple frequencies. We first simulate experimental conditions to show that as we increase the number of measured frequencies, we are able to better discriminate among different cells. Furthermore, we use the simulation to find the optimal number and value of frequencies to use to best discriminate among different cells in general. We then fabricate a microfluidic device, calibrate it with polystyrene beads, and characterize it with BA/F3 cells. With this device, we test three different activation levels of HL60 cells treated with cytochalasin D using the optimal frequency sequence obtained in simulation to determine the differences in discrimination abilities depending on the number of frequencies used. We quantify the discrimination abilities of the optimal one, two, and three frequencies by minimizing 0-1 loss.

## Microfluidics: single cell characterization by dielectrophoresis

Dielectrophoresis enables the electrical properties of single cells to be determined by application of multiple electrical frequencies. Label-free approaches for studying single cells are desirable because there is little risk of the cell being affected; dielectrophoresis is attractive for label-free characterization of the electrical properties of single cells, but depth of analysis has been limited to 1-2 frequencies, reducing ability to discriminate between different cells. Now, Alex Jaffe and Joel Voldman from the Massachusetts Institute of Technology in the US report a multi-frequency analysis approach for single cells. Following simulations to understand the most effective frequencies for cell discrimination, they experimentally demonstrate that multi-frequency analysis can identify HL60 cells treated with differing concentrations of cytochalasin D.

## Introduction

Cell-based assays in microfluidics are of significant importance, being employed for basic science as well as the diagnosis of disease^1^. Assays of single cells, as opposed to populations, is of particular interest given the widespread understanding of population heterogeneity and the importance of rare cells^2^. One class of single-cell-based assays are those that are label-free, giving them the advantage of being able to measure cellular phenotype or separate cells based on those phenotypes without altering the cell via labeling with dye, antibody, and so on^3^. Label-free assays include measurements of cell size, optical properties^4^ acoustic properties^5^, and mechanical properties^6–8^. In particular, one popular class of label-free cell-based assay examines cells’ electrical properties.

There currently are three central methods of analyzing single cells by their electrical properties: electrorotation, impedance cytometry, and dielectrophoresis^8–10^. Each method has tradeoffs in their throughput and specificity (based on the depth of analysis of each cell). Electrorotation uses a rotating electric field to induce the rotation of a particle as a result of electrical torque, where the torque and thus the rotational velocity depends on the electrical properties of the particle^11,12^. Measurement of the rotational velocity thus allows estimation of the electrical properties of cells. Electrorotation has been extended to allow for analysis of hundreds of cells at once^13^. However, acquiring a full spectrum for a single cell takes around 30 min^10,14,15^, which lowers throughput.

In comparison to electrorotation, microfluidic impedance cytometry is generally higher throughput^16,17^. It involves the continuous flow of cells through a channel where electrodes record cell impedance, often at two frequencies^18^. The utility of impedance cytometry is well exemplified in work by Morgan and colleagues^17^, where impedance cytometry was used to perform a three-part differential white blood cell count with a throughput of about 1000 cells per second. However, when not combined with other methods, such as optics and fluorescence^19^, it is typically limited the two frequencies per single cell^16^.

Dielectrophoretic (DEP) methods for discriminating single cells tend to have throughputs lower than impedance cytometry but higher than electrorotation^18^. DEP methods apply a non-uniform electric field to induce a translational DEP force on a cell. Sometimes the measurement involves a force balance between a DEP force and a fluidic drag force, yielding an observable balance position that maps a cell position to its Clausius–Mossotti (CM) factor^20–23^. In 2013 we introduced the DEP spring, in which a DEP force induced by coplanar electrodes exerts a force that is balanced by fluid drag, resulting in a well-defined balance position^23^. We used this approach to analyze cells on a single-cell basis under continuous flow. Balance positions were obtained for thousands of single cells at a given frequency and solution conductivity. These balance positions yielded estimates of the CM factors of cells. Unfortunately, the method only allowed for measuring a single frequency for each cell, limiting the depth of analysis.

Here we extend the DEP spring to measure multiple frequencies. Measurement at different frequencies allows investigation of the frequency-dependent electrical phenotype of the cells, as the measurements are obtained at frequencies that probe different parts of the cell. We call this new method the multi-frequency DEP spring. We first use stochastic simulations to understand how increasing the number of measured frequencies increases the ability to discriminate cells. Then, informed by the simulations, we develop and characterize the multi-frequency DEP spring and show its utility in characterizing cells exposed to cytoskeletal inhibitors.

## Results

We first undertook simulations to understand how measuring multiple frequencies affects cell discrimination ability and what frequencies are optimal for discriminating cells. In practical experiments, one could only observe cells at a discrete and limited set of frequencies, providing incomplete information as to the cells’ electrical properties. Therefore, we used simulations to test a large set of frequencies to indicate which subset of frequencies we should use during experiments. Given a single-shell model of a mammalian cell, considering the cytoplasm and the membrane as separate compartments, there were five dielectric parameters that identified a cell: cytoplasm permittivity and conductivity, membrane permittivity and conductivity, and radius. We could thus completely describe this model via perfect measurements of the CM factor at five independent frequencies^24^. However, all measurements had associated uncertainty, and high frequencies, which provided information as to the cytoplasmic compartment, are challenging to access. We thus wanted to determine which measurement frequencies (and how many) provided the most information about a cell when measured with some uncertainty.

### Simulation

We used a Monte Carlo simulation to create a set of cells whose properties are drawn from a distribution of cell electrical properties using continuous distributions of parameters in the single-shell model of a cell. These parameters were cell radius, cytoplasmic conductivity and permittivity, membrane (shell) conductivity, permittivity and thickness, and medium properties. We used uniform distributions of parameters across a range informed by literature (Table 1). In our model we fixed the shell thickness, due to the known thickness of the plasma membrane’s phospholipid bilayer, as well as the medium conductivity and permittivity.

**Table 1:** The values used for the parameters in the simulations

Parameters	Values
Cytoplasm conductivity	0.2↔1.2 S/m^26,27^
Cytoplasm permittivity	$20{\it{\epsilon }}_0 \leftrightarrow 80{\it{\epsilon }}_0$ ^26– 28^
Membrane conductivity	10 nS/m↔1 μS/m^26,27^
Membrane permittivity	$2{\it{\epsilon }}_0 \leftrightarrow 20{\it{\epsilon }}_0$ ^26, 27, 29^
Medium conductivity	1.5 S/m
Medium permittivity	$78.5{\it{\epsilon }}_0$
Outer radius	2.0 μm↔8.0 μm
Inner radius	1.99 μm↔7.99 μm^30^

The medium properties are held constant as they experimentally controlled. The inner radius of the cell is constant relative to the outer radius of the cell, as we assume a phospholipid bilayer thickness of 10 nm.

The underlying method of the simulation first created cells drawn from a distribution of properties and simulates their CM factors based on those properties (Supplemental Figure S1A). It then determined the optimal frequency that would differentiate most cells from one another under some assumption of the uncertainty of the measurement (Supplemental Figure S1A). In our case, we estimated the position uncertainty based on experimental balance position measurements. In the simulations, we sought to find the number of cells remaining within a set tolerance in CM factor dependent on predicted balance position (Supplemental Figure S1E) at a given frequency. The chosen frequency with the fewest cells remaining was chosen as the best first frequency. Then, using that first frequency, the algorithm searched for the best second frequency (Supplemental Figure S1B). This approach continued until including further frequencies did not further narrow the number of cells (Supplemental Figure S1C, D).

**Fig. 1:**
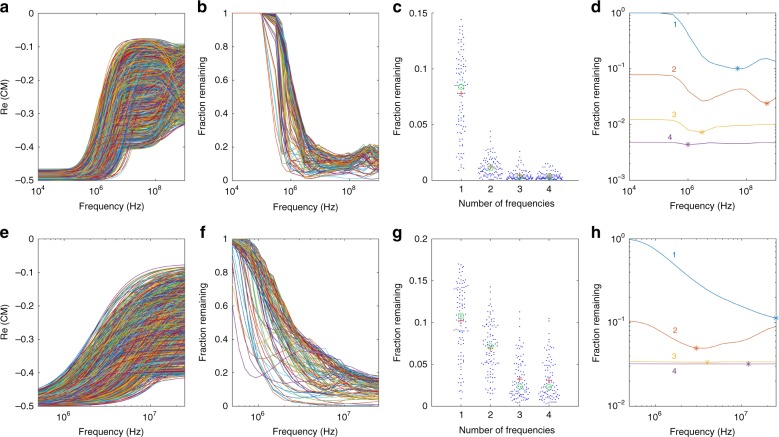
Multi-frequency simulation results. Monte Carlo simulation results for measurements across a wide (**a**–**d**) and narrow (**e**–**h**) frequency range. **a**, **e** Re[CM] factors for one simulation of 1000 randomly generated cell models. **b**, **f** Fraction of cells remaining in the threshold after balance positions at various frequencies are measured across 100 simulations. **c**, **g** Fraction of cells remaining as balance positions are measured at an increasing number of frequencies (+=mean, o = median). **d**, **h** Mean fraction of cells remaining as a function of which frequency is tested, as balance positions at increasing numbers of frequencies are measured (denoted by the number next to each line). Results for each additional frequency (2–4) are predicated on choosing the best prior frequency

As shown in Fig. 1, this simulation was executed for two frequency ranges to determine whether experimental limitations affected the optimal choice of frequencies. In one case, we simulated a large frequency range (10 kHz to 1 GHz), while in the second case we narrowed to the range explored experimentally (500 kHz to 25 MHz).

Comparing the two ranges (Fig. 1a, e), we could see that the Re[CM] spectrum had two dispersions in the wider frequency range (Fig. 1a) and only one in the narrower experimentally accessible range (Fig. 1e), consistent with the known general location of the higher-frequency dispersion. Fewer dispersions indicated that there was more independent information in the wider frequency range than in the narrower range. Examining how increasing numbers of test frequencies narrows the fraction of cells remaining in the wide experimental range (Fig. 1c, d), we saw that the ability to discern cells improves as we increased from one to four different frequencies in the sequence. In particular, measuring at one frequency (optimally chosen to be 50 MHz, Fig. 1d), left 11% of cells on average; increasing to two frequencies left only 3% of cells remaining, while four frequencies narrowed down to 0.5% of cells (Fig. 1d). Adjusting the balance position uncertainty affected the number of frequencies above which no further discrimination occurs, as expected (Supplemental Figures S2–S3).

Examining the particular optimal frequencies chosen in the wide frequency range, frequencies around the second dispersion were more informative than lower frequencies as the frequencies around the second dispersion had the high variability in CM (Fig. 1a) and the balance position was strongly sensitive to Re[CM] when Re[CM] was close to zero (Figure S1E). After that, the optimal frequencies were around the first dispersion as CM factor spectra around the second dispersion were already narrowed down from the first optimal frequency. The fourth frequency only provided 0.3% additional discrimination ability, and the particular choice of frequency value was not very sensitive (0.05% change in fraction of cells remaining across the frequency range).

Turning to the narrower, more easily accessible experimental range (Fig. 1e–h), we saw a similar decrease in the fraction of cells remaining as additional frequencies were measured (Fig. 1g). Consistent with the wide frequency range, the optimal first frequency was the highest-accessible frequency, in this case 25 MHz. We also saw that the ability to discriminate saturated at ~4% cells remaining after three frequencies, worse than in the wider frequency range, because not all cell properties were accessible at the lower frequency range. In particular, the third frequency choice was not critical; there was a 0.1% variation in the fraction of cells remaining across the frequency range.

We chose an optimal frequency sequence to use for experiments by observing which frequency sequence was most commonly found to be the optimal three-frequency combination in our simulations across the experimental range. Based on these results, the optimal frequency combination was 1.2, 2, and 25 MHz, which were the frequencies used for experiments. Since the simulation was run 100 times, the optimal three-frequency combination varied for different simulation runs. For this reason, average optimal frequencies for each numbered frequency (1–4) in Fig. 1d, h differ from the optimal frequency combination found.

### Multi-frequency DEP spring

**Fig. 2:**
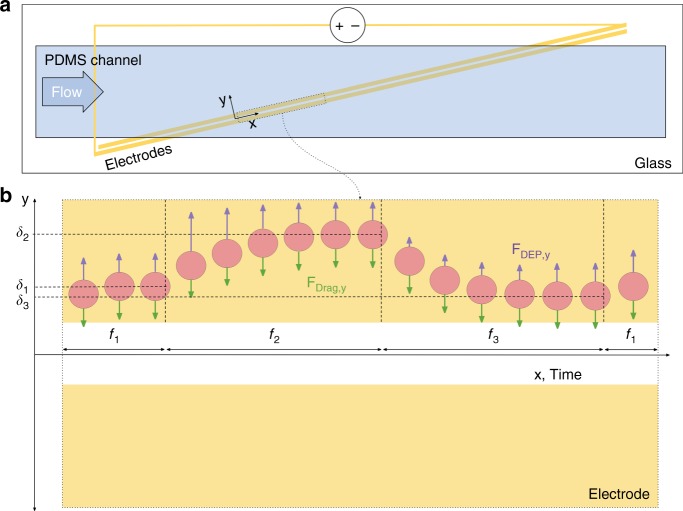
Multi-frequency DEP spring overview. **a** Schematic of the channel with slanted electrodes. **b** Schematic of a single cell experiencing the DEP spring at multiple frequencies (*f*_1_, f_2_, and *f*_3_) at different points in time, where they experience a balance between the *y*-directed DEP force (*F*_DEP,*y*_) and the *y*-directed drag force (*F*_Drag,*y*_) and arrive at balance positions δ_1_, δ_2_, and δ_3_, respectively. In this instantiation three frequencies repeat. The center of the electrodes defines the origin of the *y*-axis

Experiments were run using a microfluidic DEP spring^23^ device consisting of a polydimethylsiloxane (PDMS) channel atop a glass substrate containing coplanar electrodes (Fig. 2). Cells flowed through the channel and experience a negative DEP force when they encountered two angled electrodes on the floor of the chamber (Fig. 2a and Supplemental Movie). This DEP force was counteracted by the fluid drag force. When these forces were of equal magnitude, the cell would reach a balance position, which was dependent on the applied frequency, the Re[CM] factor of the cells, cell size, electric field intensity, and so on. The balance position is given by:

$$\delta = q_R^{ - 1}\left( {\frac{{3\eta\,{\mathrm{sin}}\,\theta \left[ {\frac{{6Q}}{{wh^3}}\left( {h - R} \right)} \right]}}{{R\varepsilon _{\mathrm{m}}{\mathrm{Re}}\left[ {{\mathrm{CM}}} \right]V_{{\mathrm{RMS}}}^2p\left( {f,\sigma _{\mathrm{m}}} \right)}}} \right),$$

where *q*_*R*_^−1^ reflects the dependence of the DEP force on position (ref. 23), *η* is the medium viscosity, *θ* is the angle between the electrodes and the fluid flow in the channel, *w* is the channel width, *h* is channel height, *R* is the cell radius, *ε*_m_ is the medium permittivity, *V*_RMS_ is the root-mean-square voltage across the electrodes, and *p*(*f*, *σ*_m_) a normalization factor that corrects for any drop at the electrode solution interface within the channel that depends on frequency and media conductivity *σ*_m_.

In the multi-frequency DEP spring, the frequencies changed according to a predetermined sequence (shown as *f*_1_, *f*_2_, *f*_3_, *f*_1_, etc. in Fig. 2b), which changed the balance position by changing the Re[CM]. The cells attained the new balance position with some settling time. By visually tracking the cells across the field of view (Fig. 2b) and correlating the image stack timestamps to the frequency sequence timestamps, we could correlate balance position to applied frequency.

### BA/F3 balance position verification

**Fig. 3:**
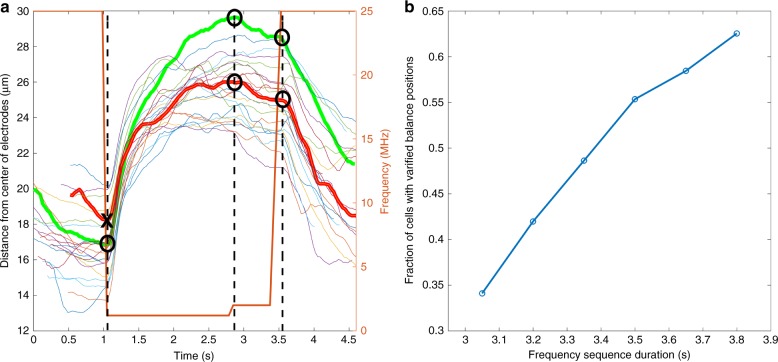
Balance position measurements. **a** Overlaid measured trajectories of 23 BA/F3 cells, along with the frequency the cells are experiencing as a function in time (bold orange). The bolded green trajectory is an example of a cell that properly attains all three balance positions. The bolded red trajectory is an example of a cell that does not properly attain all three balance positions. The black circles indicate validated balance positions while the black X shows an invalidated balance position. The vertical dashed lines indicate transitions in frequency where balance positions are measured. **b** Fraction of valid balance positions measured as the overall time for the frequency sequence changes

To validate the ability to measure balance positions at multiple frequencies and understand the limits of the measurements, we undertook experiments with BA/F3 cells. Cells were subjected to sequences of measurement frequencies and their positions were measured (Fig. 3). We defined a valid balance position if the cell stayed within 10% of the average difference in balance positions between one frequency and the next frequency in the final 250 ms at a given frequency. If this criterion was met, the balance position was defined to be the final distance measurement from the center of the electrodes at a given frequency.

Figure 3a shows, among several other cell trajectories, an example of a cell trajectory that properly attained all three balance positions and an example of a cell trajectory that did not do so. The frequency sequence used was 25 MHz for 1300 ms, followed by 1.2 MHz for 1800 ms, followed by 2 MHz for 700 ms, and then repeated. Due to the similarity in balance positions between 1.2 and 2 MHz, this transition took less time and could afford a shorter duration at 2 MHz. The differences in balance positions between 25 and 1.2 MHz were the largest, causing 1.2 MHz to necessitate the longest duration. We found that ~63% of cells properly attained all three balance positions, while the remainder failed to properly attain at least one of three balance positions. We then computationally examined how decreasing the time at each frequency would affect the fraction of cells that attain valid balance positions (Fig. 3b); note that this metric differs from the fraction of cells unable to be discriminated referred to in Fig. 1. As expected, decreasing the total frequency sequence duration from 3.8 to 3.05 s decreased the fraction of valid balance positions to 40%. However, decreasing the duration would allow more balance positions to be measured, assuming camera field of view and flow rate would be kept constant. Overall, then, we found that we were able to reliably measure three frequencies, with the ability to increase the number of measured frequencies, if desired, by altering experimental parameters.

### Cell discrimination

Finally, we sought to apply the multi-frequency DEP spring to the problem of distinguishing closely related cell states. In particular, we measured the electrical properties of HL60 cells under exposure to different concentrations of cytochalasin D (CytoD), a drug that blocks actin cytoskeleton polymerization. We chose this treatment to examine whether the known effects of the drug on deformability also translated to any changes in electrical properties.

**Fig. 4:**
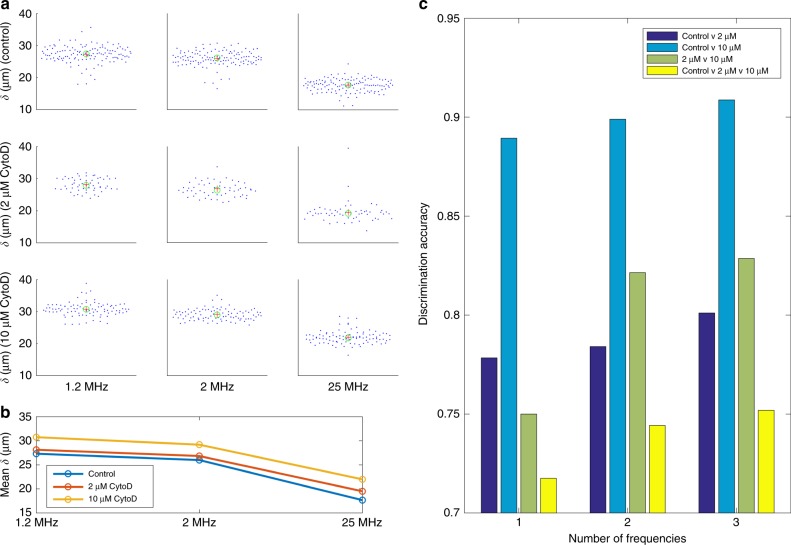
Multi-frequency balance positions of HL60 cells. **a** Balance positions for 262 HL60 cells across treatment condition and frequency (+=mean, o = median). **b** Balance position means at all three frequencies separated by treatment condition. **c** Discrimination accuracies as the number of measured frequencies changes. The yellow bars signify classification into one of three possible classes, whereas the other colors signify classification into one of two possible classes

We measured multi-frequency balance positions of 262 cells exposed to two concentrations of CytoD (along with control). To avoid any nonspecific changes due to changes in the cell size, we adjusted for the cell size variations (measured optically) using Eq. 1. In addition, we adjusted for any frequency dependence in the applied field (due to electrode polarization or lead inductance) by making control measurements with polystyrene beads (Supplemental Figure S4), and using those balance positions to compensate. Figure 4a presents scatter plots of the measurements. We saw that as cells are treated with increasing concentrations of CytoD, their size-corrected population-average balance positions increased in a dose-dependent manner (Fig. 4b), consistent with an increase in the nDEP force and suggesting a concomitant increase in Re[CM].

To explore the utility of measuring multiple frequencies for distinguishing cells, we trained a 0-1 loss classifier to discriminate between the cells given the three derived Re[CM] factors for each cell. We see in Fig. 4c that the discrimination ability when classifying between two populations or among three populations improved when increasing the number of frequencies at which we measured balance positions. We also observe that, as expected, it was easiest to distinguish control from 10 μM CytoD. Furthermore, all 12 discrimination accuracies shown correspond to statistically significant discriminations with paired-sample *t* test *p* values for each ranging from as high as 0.0012 to as low as 3.8e−14 (*p* < 0.05 is significant).

## Discussion

Our goal in introducing the multi-frequency DEP spring was to obtain more information about cells’ electrical properties while maintaining throughput. We showed in our simulations that the wider the frequency range is and the more dispersions it has, the more frequencies become useful in discriminating cells. We then defined a metric for assessing the transition from frequency to frequency using BA/F3 cells in order to experimentally ascertain cell balance positions, yielding CM factor values at certain frequencies. Finally, we used three frequencies on single HL60 cells of three different subpopulations to show that as more frequencies are employed to identify the HL60 cells, discrimination accuracy increases.

Considering the simulation we were able to find an optimal number of frequencies to use given both an experimental range and a wider theoretical range. For the former, we observed that three frequencies maximized the information provided, while in the wider range of frequencies, four frequencies substantially increases information. From a theoretical standpoint, we can credit this to being able to access the higher-frequency dispersion in the Re[CM] spectrum with the wider frequency range than the experimental range. From a physical standpoint, we can state that a wider range of frequencies will reach a wider range of compartments and their electrical parameters^24^. In both cases, the precision of the measurements (uncertainty in balance position, in this case) affected the ability to discriminate cells. Currently, the balance position uncertainty does not limit the ability to discriminate cells.

When we consider which frequencies in the experimental range to use, the results consistently indicate that a frequency high in the range and a frequency low in the range should be included. This is not surprising given that in the single-cell model the high frequencies probe different parameters than the low frequencies. However, the particular optimal frequencies varied with each simulation run, as expected for a stochastic model.

Results with Cyto-treated cells highlight the improved information content derived when measuring cells at multiple frequencies. Here we see increasing accuracy with increasing number of frequencies, regardless of the comparison set. Although we focused experimentally on measuring three frequencies, informed by the simulation results, it might be useful experimentally to measure cells at additional frequencies. This is because cells’ electrical properties do not necessarily follow the single-shell model exactly. The upper bound on the number of balance positions that one can measure are based on the flowrate and the imaging field-of-view. Decreasing flow rate would increase the residence time and thus the ability to measure additional frequencies, though it could also increase the time needed to achieve a particular balance position. Increasing the microscope field-of-view would also increase the number of frequencies that could be measured, although typically with a tradeoff in spatial resolution and thus potentially increased uncertainty in the balance position.

On the topic of uncertainty, one can observe that measuring cells of the same population with our device can produce noticeably different balance positions. Although true biophysical differences among cells within the same population would yield different balance positions, measurement error also plays a role in adding uncertainty to the balance position measurement. Causes of measurement error include variations in channel height along the length of the channel within the field of view causing an unintended *x*-axis dependence of balance position, as well as variations in the electrodes producing the electric field acting on the cells. Improved control of such factors would help to increase discrimination accuracy. In addition, one can leverage repeated observations of the cell as it traverses the field of view to develop a better estimate of the balance position.

The same device was used for all experiments mentioned. This allowed us to maintain relatively constant device properties, such as channel width, channel height, and electric field at a given frequency, across experiments. These device properties are factors in determining the CM factors of the cells traveling through the device channel and can vary from device to device if separate devices are not fabricated in exactly the same manner. Therefore, using separate devices for the same experiment would add uncertainty to our results.

Comparing this method to other methods of discriminating single cells by their electrical properties, we find that our throughput (up to ~1 cell/s) exceeds that of electrorotation, where the typical maximum throughput is ~5 cells/h^14,15^, which is considerably lower than our maximum throughput, although the depth of analysis is greater (~20 frequencies/cell^11^). Regarding impedance cytometry, we see that the throughput is much higher (~1000 cells/s), but the typical maximum number of frequencies per cell is two, while we have shown our method yielding three data points per cell in the form of balance positions, and this can be easily increased. Finally, comparing to other DEP-based electrical cell measurement techniques, most single-cell DEP methods measure cell properties at a single frequency^20^; we have shown utility in measuring more frequencies per cell as a means of increasing cell discrimination ability.

Despite showing utility with our method, we note that our current throughput is indeed modest. Several approaches exist to increase throughput, such as by increasing cell concentration and using image tracking methods to keep track of all of the cells in the field of view. Furthermore, we could increase flow rate while also increasing the voltage peak-to-peak amplitude between the electrodes to assure balance positions would still be attained. Field strength could also be increased, allowing a higher flow rate, by putting electrodes on top of the channel as well as on the bottom of the channel, as we have done in other work^25^.

An important next step in our experimentation would be showing our ability to discriminate cells in a mixture. The experiments presented here have discriminated cells in pure populations. In order to discriminate within mixed populations, we could use a label to provide ground-truth information as to the cell identity. As long as cells are far enough apart such that they do not interact electrically or hydrodynamically, measuring in mixed populations should be straightforward.

## Conclusions

Here we show through simulation and experiments that measuring electrical properties of cells at multiple frequencies for single cells increases our ability to discriminate them. We first quantify how this ability improves through simulation of testing multiple frequencies per cell, improving by showing fewer and fewer CM factor spectra within tolerance thresholds as we incrementally increase the amount of frequencies per cell from one to four. We then establish what it means to successfully attain balance positions when transitioning from frequency to frequency within the experimental multi-frequency DEP spring, showing that there is a rough limit to how many frequencies can be tested on a single cell. Finally, we show consistent increases in accuracy when discriminating HL60 cells treated with different concentrations of CytoD when increasing from one to two and two to three measured frequencies per cell using the optimal frequencies obtained from our simulation.

## Materials and methods

### Simulation

Simulations were performed using MATLAB 2016a. The Monte Carlo simulation of the intrinsic parameters was varied with a uniform distribution across the given ranges (Table 1). The simulation was run 100 times with 1000 simulated cells CM factor spectra per run. For each run, a cell CM factor spectrum was chosen at random, and a tolerance in the CM factor was estimated based on the experimentally measured balance position uncertainty. We used a position uncertainty of 0.5 microns, which was ~2× the standard deviation of the noise in the experimentally measured balance positions. When a given frequency was tested, certain CM factor spectra would be similar enough at that frequency to the chosen CM factor spectrum to remain within the tolerance. However, several other CM factor spectra will not. Whichever frequency in the spectrum resulted in the fewest neighboring cell CM factor spectra within the CM factor tolerance was chosen to be the optimal frequency. That optimal frequency was then held constant and the process repeated to select the second optimal frequency. Once the second optimal frequency is selected, the first two frequencies are held constant and the process repeated to select the third optimal frequency, and so on.

### Device fabrication

The device channel was made with Sylgard 184 PDMS, using a cross-linker-to-elastomer ratio of 1:10, and cleaned with isopropanol. The Ti/Au device electrodes were fabricated on a glass substrate using standard microfabrication methods and were cut with a dice saw and cleaned with acetone, methanol, and isopropanol. Three holes for input ports and three output ports were punched into the PDMS channel device. The device channel was attached to the device electrodes using plasma from a Harrick plasma cleaner/sterilizer chamber (model PDC-32G), creating a three dimensional channel with the sides and top being the PDMS and the bottom being the gold electrodes on the glass substrate.

### Cell culture

BA/F3 cells were cultured from frozen stalk at −80 °C. The media used were RPMI media with penicillin/streptomycin (1×), fetal bovine serum (20%), and l-glutamine (2 mM). The cells were passaged at a 1:10 ratio every 5 days. HL60 cells were similarly cultured in Dulbecco's modified Eagle's media with penicillin streptomycin (1×), bovine calf serum (20%), and l-glutamine (2 mM). Both the BA/F3 and HL60 cells typically have over 90% viability.

### Bead preparation

Ten microns (10.269 ± 0.502 μm) of carboxylate-modified polystyrene beads from Polysciences Inc. (Warrington, PA, USA) were prepared for experiments by diluting in phosphate-buffered saline (PBS) at a 1:100 ratio.

### Cell and bead experiments

Flow was controlled by two Chemyx Fusion 200 syringe pumps. Before each experiment, the device was primed with 1% bovine serum albumin in PBS by passing it through the device channel at a flow rate of 20 μL/min for 30 min. Meanwhile, 1 mL cells in media were centrifuged down at 1000 RPM for 5 min and the supernatant media were replaced with PBS. PBS was pumped through two of the input ports, while PBS with cells at the desired concentration (10^6^ cells/ml) was pumped through the third input port with a total flow rate of 0.6 µL/min. This total flow rate is kept constant throughout the entirety of the experiment. Images of cells were obtained by a LAVision Imager QE camera coupled to a Zeiss Imager.M1m microscope with a ×10 objective lens and brightfield illumination. Voltages at frequencies <15 MHz were applied via an Agilent 33220A function generator, while those >15 MHz were applied by a TGR1040 RF signal generator and amplified by a TVA-R5-13 RF power amplifier. The microscope, the camera, and both function generators had automated control from a MATLAB GUI. After the experiment’s end, the device was cleaned with PBS at a flow rate of 40 μL/min for 5 min, and then distilled water at a flow rate of 100 μL/min for 1 min. The device was then purged with air for drying for 5 min and then stored.

### Data processing

During each experiment image stacks were collected by the MATLAB GUI. The image stacks were used as inputs to a particle detection script which outputted detected particles as data structures by means of a time domain median image filter and pixel intensity thresholding. The detected particles were tracked using a particle tracking script, which tracks the particles for the duration of time they are in the field of view of the microscope, applies a four-point moving average time domain filter to each particle, and defines balance positions as the final *y*-position of a particle at a certain frequency. Validated particles and their trajectories were then stored as MATLAB data files for analysis. The validated balance positions for different populations were classified by optimizing a weight vector through minimizing 0-1 classification loss. After this optimization, the discrimination accruacy was calculated via the formula:

$$\begin{array}{l}{\rm{Discrimination}}\,{\rm{accuracy}}\\ = \frac{{\rm{Amount}}\,{\rm{of}}\,{\rm{Cells}}\,{\rm{Tested}} - {\rm{Amount}}\,{\rm{of}}\,{\rm{Errors}}\,{\rm{Made}}}{{{\rm{Amount}}\,{\rm{of}}\,{\rm{Cells}}\,{\rm{Tested}}}}.\end{array}$$

## Electronic supplementary material

Supplementary Info Readme

Supplementary Information Document

HL60 Cells Traveling Through Channel

## References

[1] Sackmann EK, Fulton AL, Beebe DJ (2014). The present and future role of microfluidics in biomedical research. Nature.

[2] Zare RN, Kim S (2010). Microfluidic platforms for single-cell analysis. Annu. Rev. Biomed. Eng..

[3] Minor LK (2008). Label-free cell-based functional assays. Comb. Chem. High. T Scr..

[4] Fang Y (2006). Label-free cell-based assays with optical biosensors in drug discovery. Assay Drug Dev. Technol..

[5] Petersson F, Åberg L, Swärd-Nilsson AM, Laurell T (2007). Free flow acoustophoresis: microfluidic-based mode of particle and cell separation. Anal. Chem..

[6] Cross SE, Jin YS, Rao J, Gimzewski JK (2007). Nanomechanical analysis of cells from cancer patients. Nat. Nanotechnol..

[7] Hur SC, Henderson-MacLennan NK, McCabe ER, Di Carlo D (2011). Deformability-based cell classification and enrichment using inertial microfluidics. Lab Chip.

[8] Morgan H, Sun T, Holmes D, Gawad S, Green NG (2006). Single cell dielectric spectroscopy. J. Phys. D.

[9] Voldman J (2006). Electrical forces for microscale cell manipulation. Annu. Rev. Biomed. Eng..

[10] Mansor MA, Ahmad MR (2015). Single cell electrical characterization techniques. Int J. Mol. Sci..

[11] Yang J (1999). Dielectric properties of human leukocyte subpopulations determined by electrorotation as a cell separation criterion. Biophys. J..

[12] Eppmann P, Gimsa J, Prüger B, Donath E (1996). Dynamic light scattering from oriented, rotating particles: a theoretical study and comparison to electrorotation data. J. Phys. III.

[13] Hölzel R (1997). Electrorotation of single yeast cells at frequencies between 100 Hz and 1.6 GHz. Biophys. J..

[14] De Gasperis G, Wang X, Yang J, Becker FF, Gascoyne PR (1998). Automated electrorotation: dielectric characterization of living cells by real-time motion estimation. Meas. Sci. Technol..

[15] Cristofanilli M (2002). Automated electrorotation to reveal dielectric variations related to HER-2/neu overexpression in MCF-7 sublines. Clin. Cancer Res..

[16] Sun T, Morgan H (2010). Single-cell microfluidic impedance cytometry: a review. Microfluid. Nanofluid..

[17] Holmes D (2009). Leukocyte analysis and differentiation using high speed microfluidic single cell impedance cytometry. Lab Chip.

[18] Chen J (2015). Microfluidic impedance flow cytometry enabling high-throughput single-cell electrical property characterization. Int. J. Mol. Sci..

[19] Spencer D, Elliott G, Morgan H (2014). A sheath-less combined optical and impedance micro-cytometer. Lab Chip.

[20] Pethig R (2010). Dielectrophoresis: status of the theory, technology, and applications. Biomicrofluidics.

[21] Huang Y, Wang XB, Becker FF, Gascoyne P (1997). Introducing dielectrophoresis as a new force field for field-flow fractionation. Biophys. J..

[22] Davis JM, Giddings JC (1986). Feasibility study of dielectrical field-flow fractionation. Sep. Sci. Technol..

[23] Su HW, Prieto JL, Voldman J (2013). Rapid dielectrophoretic characterization of single cells using the dielectrophoretic spring. Lab Chip.

[24] Morgan, H. & Green, N. G. *AC Electrokinetics* (Research Studies Press, Philadelphia, PA, 2003).

[25] Prieto JL (2016). Monitoring sepsis using electrical cell profiling. Lab Chip.

[26] Markx GH, Davey CL (1999). The dielectric properties of biological cells at radiofrequencies: applications in biotechnology. Enzyme Microb. Technol..

[27] Davey, C. L. & Kell, D. B. *The Low-Frequency Dielectric Properties of Biological Cells. Bioelectrochemistry of Cells and Tissues* 159–207 (Springer, Berlin, 1995).

[28] Gimsa J, Müller T, Schnelle T, Fuhr G (1996). Dielectric spectroscopy of single human erythrocytes at physiological ionic strength: dispersion of the cytoplasm. Biophys. J..

[29] Cevc G (1990). Membrane electrostatics. BBA-Rev. Biomembr..

[30] Andersen OS, Koeppe RE (2007). Bilayer thickness and membrane protein function: an energetic perspective. Annu. Rev. Biophys. Biomol. Struct..

