# Supplementary material for: Multi-frequency dielectrophoretic characterization of single cells
Source: Microsyst Nanoeng. 2018 Sep 10;4:23. doi: 10.1038/s41378-018-0023-4 (PMC6220158; doi:10.1038/s41378-018-0023-4)
Supplement: Supplementary file 1 — Supplementary Info Readme [file 41378_2018_23_MOESM1_ESM.docx]

Supplementary Information Readme

The supplementary information for this paper consists of a Word document consisting of six supplemental figures (MultifrequencyDEPSingleCell Supplementary Information.docx) and a Quick Time file showing HL60 cells traveling through the channel of our device (supplementary_video_1.mov).
