# Supplementary material for: Multi-frequency dielectrophoretic characterization of single cells
Source: Microsyst Nanoeng. 2018 Sep 10;4:23. doi: 10.1038/s41378-018-0023-4 (PMC6220158; doi:10.1038/s41378-018-0023-4)
Supplement: Supplementary file 2 — Supplementary Information Document [file 41378_2018_23_MOESM2_ESM.docx]

# Multi-frequency Dielectrophoretic Characterization of Single Cells - Supplemental Information


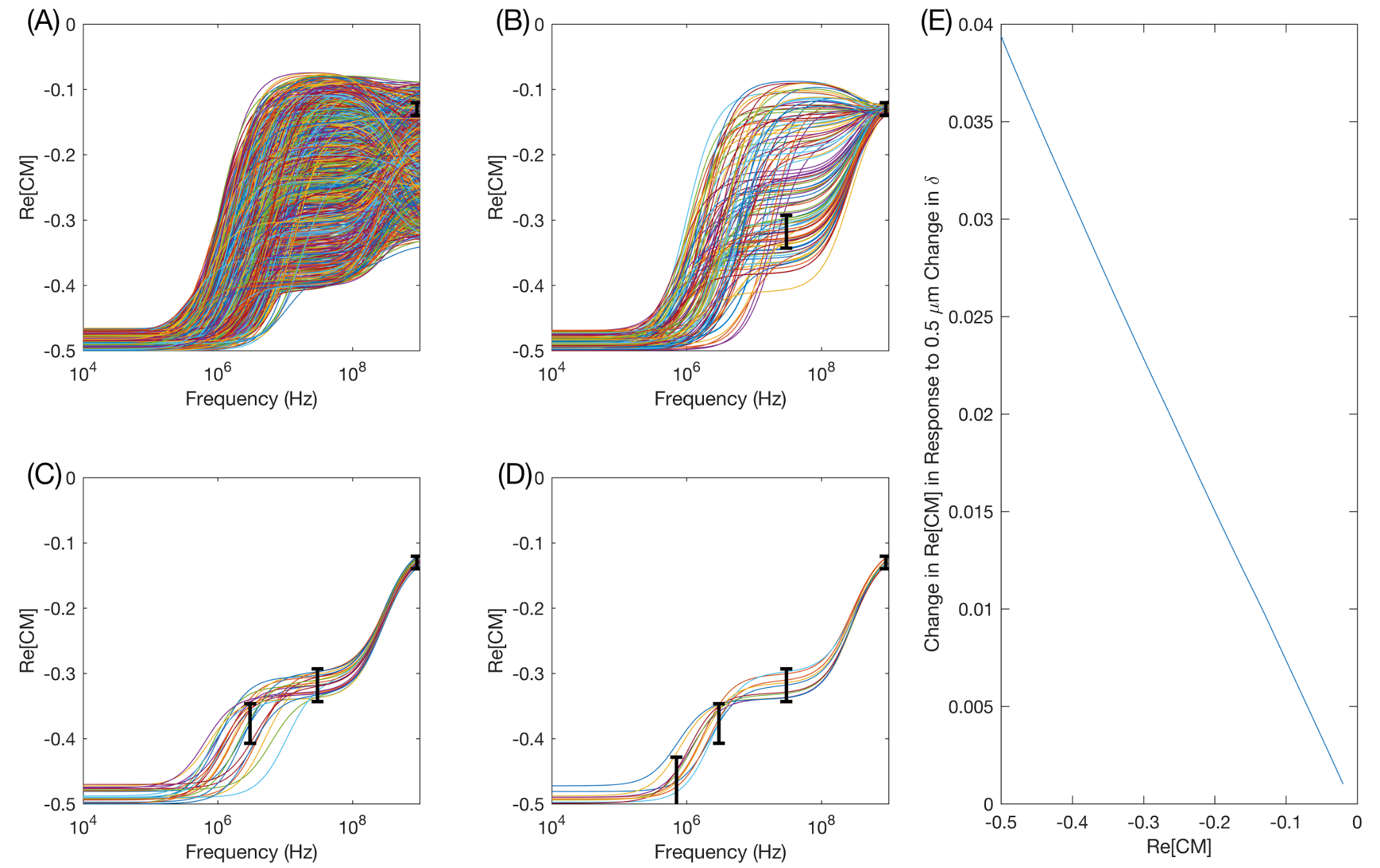


**Supplemental Figure S1: Simulation overview.** (A) Simulated CM factors for 1000 cells and the first selected optimal frequency (~900 MHz, threshold marker). The balance position uncertainty of 0.5 microns translates into an equivalent tolerance in CM factor measurement. (B) Simulated CM factors for cells still remaining after measurement of the first frequency, along with the second selected optimal frequency. (C-D) Simulated CM factors for cells remaining after measurement of the second (C) and third (D) frequency, along with the optimal third (C) and fourth (D) frequencies. (E) shows the sensitivity of Re[CM] to position uncertainty, i.e., how a 0.5 μm change in balance position δ ($\Delta\delta=0.5 \mu m)$changes Re[CM] at different Re[CM].


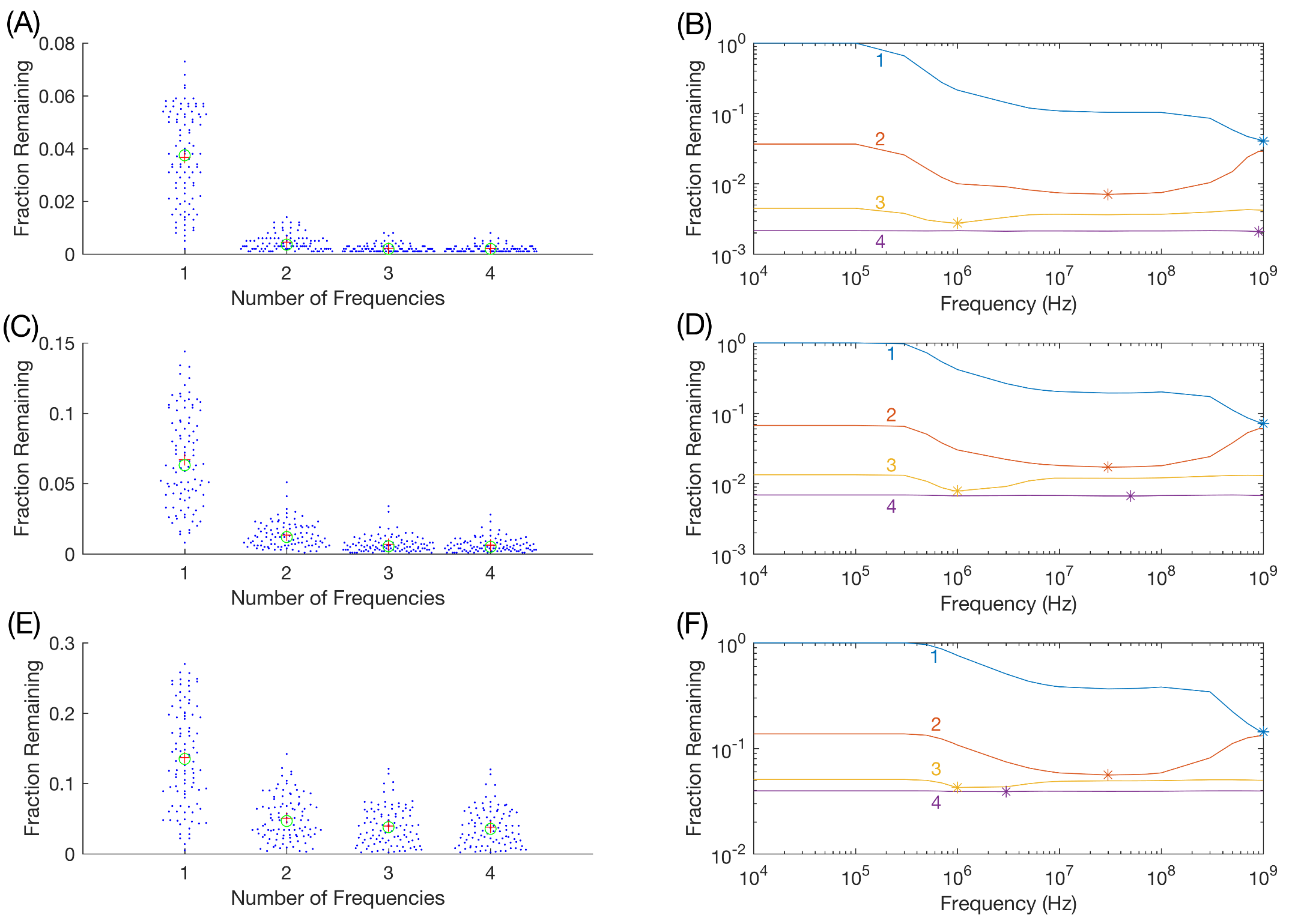


**Supplemental Figure S2: Effect of measurement uncertainty on discrimination ability in the wide frequency range.** (A,C,E) Fraction of cells remaining as balance positions are measured at an increasing number of frequencies (+ = mean, o = median) with balance position uncertainty of 0.25 microns (A), 0.5 microns (C), and 1 micron (E). (B,D,F) Mean fraction of cells remaining as a function of which frequency is tested, as balance points at increasing numbers of frequencies are measured (# of frequencies denoted on the plots), with balance position uncertainty of 0.25 microns (B), 0.5 microns (D), and 1 micron (F). Results for each additional frequency (2 to 4) are predicated on choosing the best prior frequency.


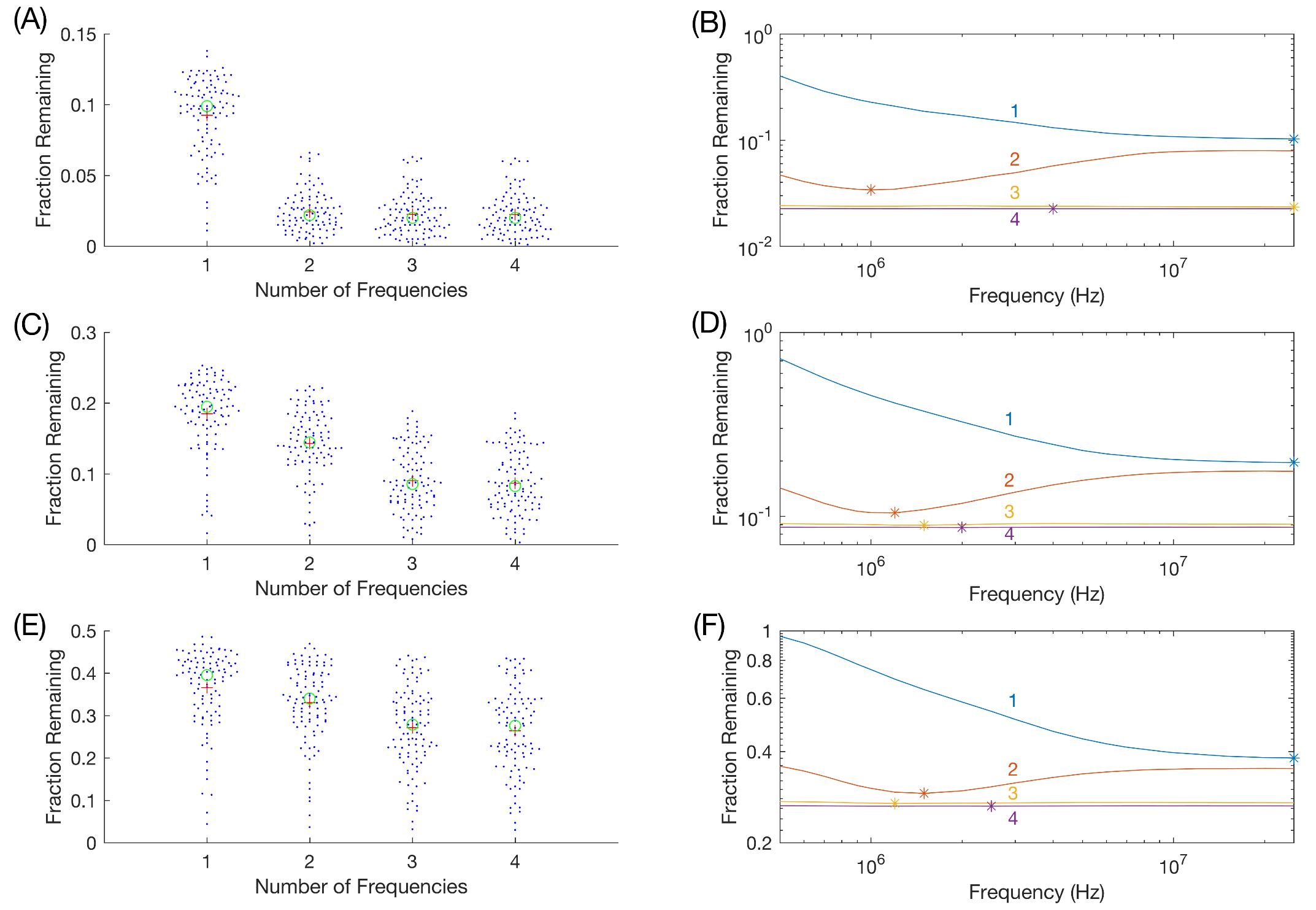


**Supplemental Figure S3: Effect of measurement uncertainty on discrimination ability in the experimental frequency range.** (A,C,E) Fraction of cells remaining as balance positions are measured at an increasing number of frequencies (+ = mean, o = median) with balance position uncertainty of 0.25 microns (A), 0.5 microns (C), and 1 micron (E). (B,D,F) Mean fraction of cells remaining as a function of which frequency is tested, as balance points at increasing numbers of frequencies are measured (# of frequencies denoted on the plots), with balance position uncertainty of 0.25 microns (B), 0.5 microns (D), and 1 micron (F). Results for each additional frequency (2 to 4) are predicated on choosing the best prior frequency.


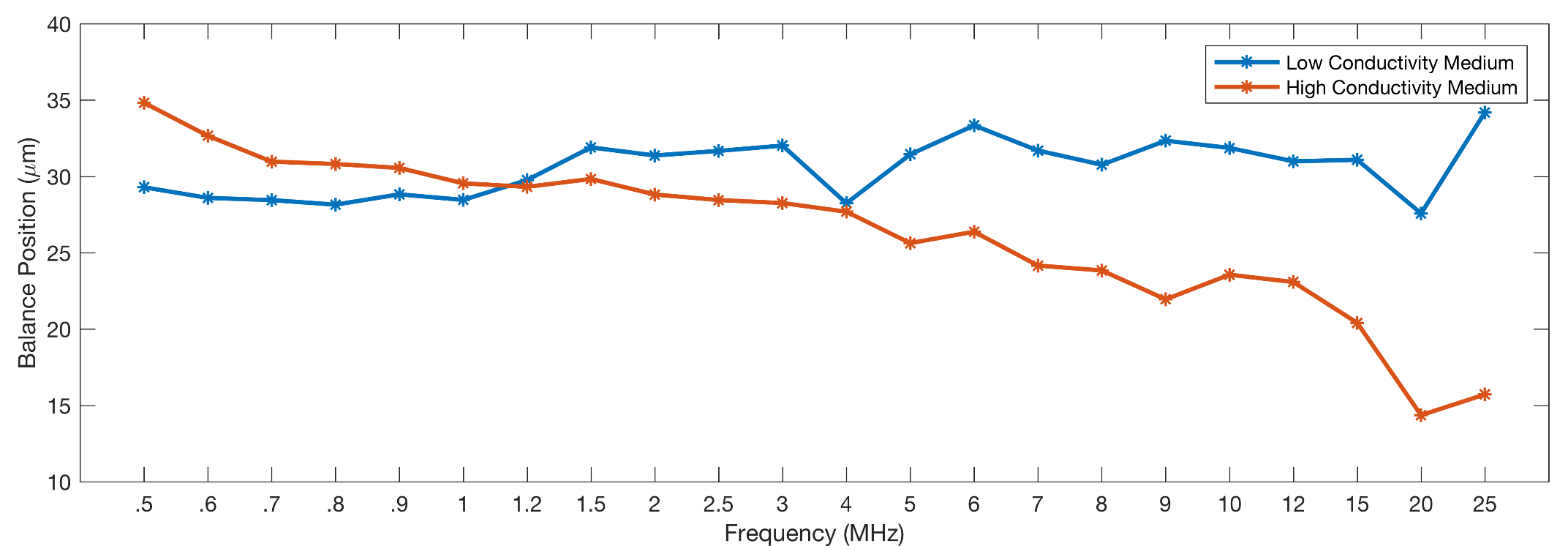


**Supplemental Figure S4: Polystyrene Bead Calibration** Balance points of 10 micron polystyrene beads in high-conductivity PBS solution (red) and low conductivity sucrose solution (bottom) while varying the input frequency. All tested frequencies were given an amplitude of 8.0 VPP except for 25 MHz which was given 12.0 VPP.


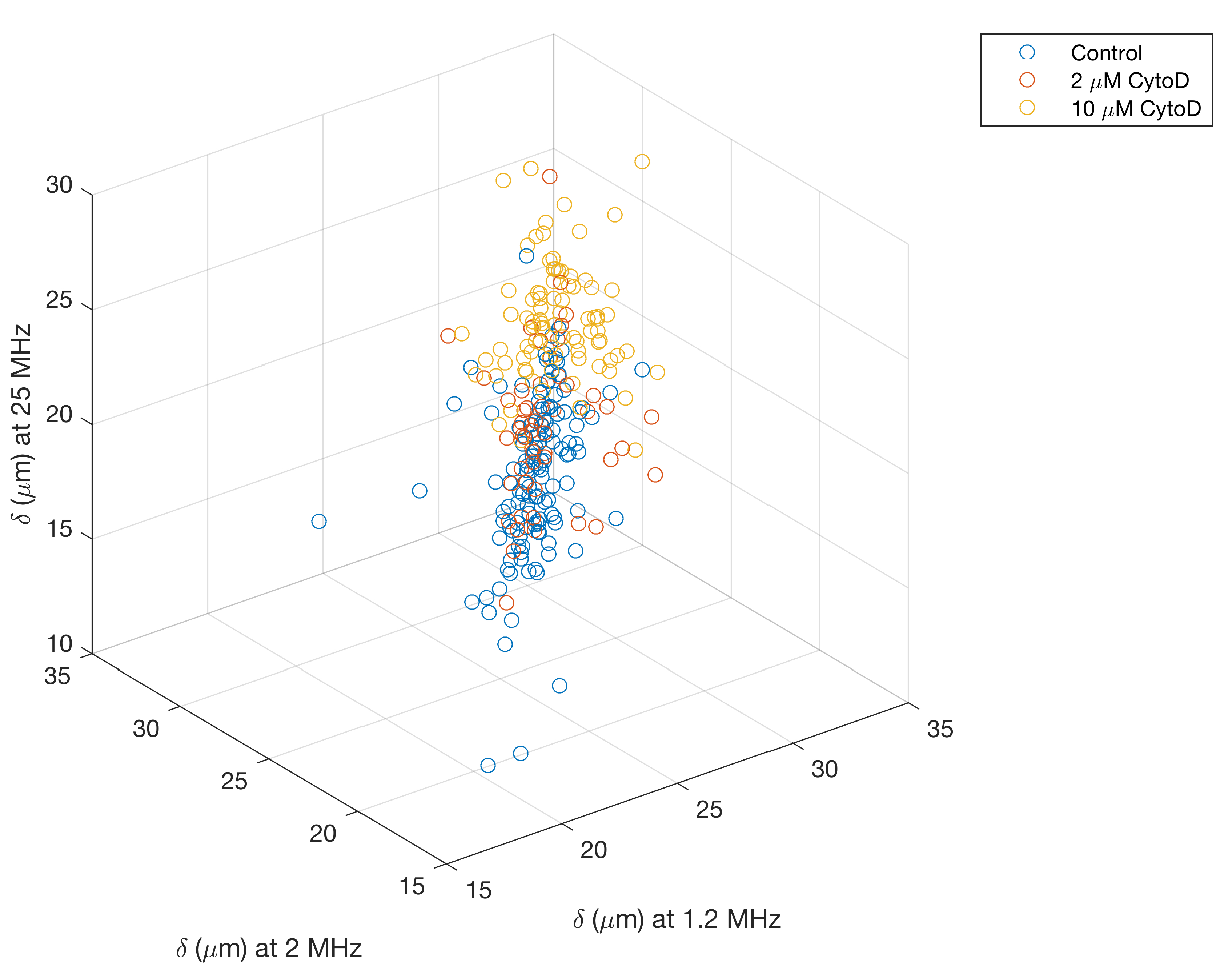


**Supplemental Figure S5: 3-D Scatter Plot of Balance Positions** Each HL60 cell tested is represented by a circle on the 3-D scatter plot with each of the three coordinates given by a balance position (ẟ)

at each of the three frequencies. The circles are colored blue for the control population, red for the population treated with 2 μM Cytochalasin D, and yellow for the population treated with 10 μM Cytochalasin D.


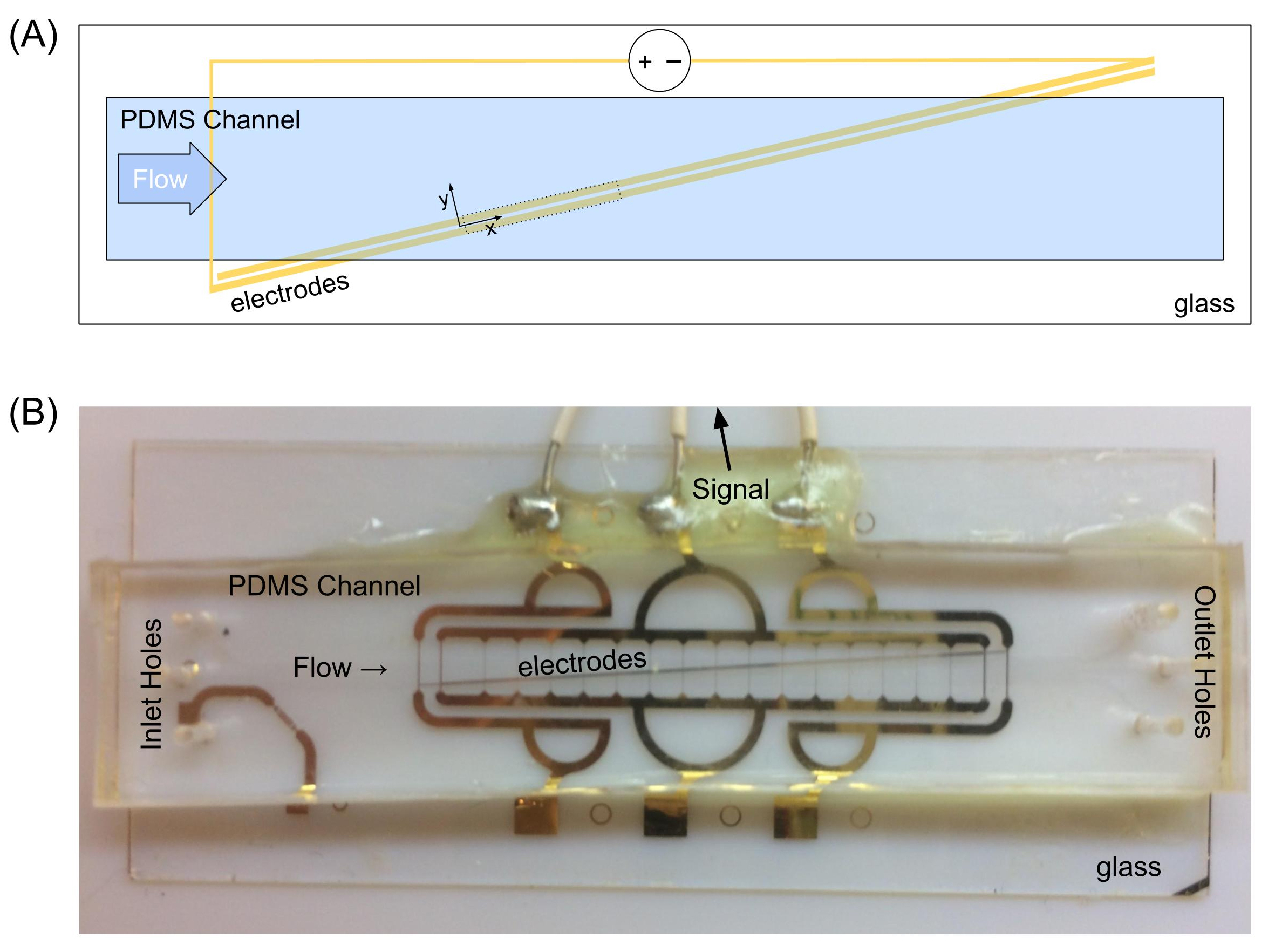


**Supplementary Figure S6: IDS Device Diagram and Labeled Picture** (A) Replicate of the device diagram shown in Figure 2A of the main text. (B) Labeled image of the device. The voltage symbol in (A) is analogous to the “Signal” text in (B) and represents the generation of an AC signal across the electrodes. The “electrodes” labels in (A) and (B) correspond to the electrodes over which cells travel and experience DEP forces. The “Flow” labels in (A) and (B) correspond to the opening in the PDMS channel through which cells flow. The “Inlet Holes” and “Outlet Holes” labels in (B) correspond to where cells and buffer enter exit the channel opening. The length of the device is 5 cm, the channel width is 2 mm, and the channel height is 20 μm.
